# Insight into the Structure and Properties of Novel Imidazole-Based Salts of Salicylic Acid

**DOI:** 10.3390/molecules24224144

**Published:** 2019-11-15

**Authors:** Inês C. B. Martins, Dominik Al-Sabbagh, Klas Meyer, Michael Maiwald, Gudrun Scholz, Franziska Emmerling

**Affiliations:** 1 Federal Institute for Materials Research and Testing (BAM), Richard Willstätter-Str- 11, 10249 Berlin, Germany; Ines-Catarina.Batista-Martins@bam.de (I.C.B.M.); Dominik.Al-Sabbagh@bam.de (D.A.-S.); Klas.Meyer@bam.de (K.M.); Michael.Maiwald@bam.de (M.M.); 2 Department of Chemistry, Humboldt-Universität zu Berlin, Brook-Taylor-Str. 2, 12489 Berlin, Germany; Gudrun.Scholz@rz.hu-berlin.de

**Keywords:** salicylic acid, imidazole, salts, powder X-ray diffraction, ssNMR, DFT

## Abstract

The preparation of new active pharmaceutical ingredient (API) multicomponent crystal forms, especially co-crystals and salts, is being considered as a reliable strategy to improve API solubility and bioavailability. In this study, three novel imidazole-based salts of the poorly water-soluble salicylic acid (SA) are reported exhibiting a remarkable improvement in solubility and dissolution rate properties. All structures were solved by powder X-ray diffraction. Multiple complementary techniques were used to solve co-crystal/salt ambiguities: density functional theory calculations, Raman and ^1^H/^13^C solid-state NMR spectroscopies. In all molecular salts, the crystal packing interactions are based on a common charged assisted ^+^N-H_(SA)_⋯O^−^_(co-former)_ hydrogen bond interaction. The presence of an extra methyl group in different positions of the co-former, induced different supramolecular arrangements, yielding salts with different physicochemical properties. All salts present much higher solubility and dissolution rate than pure SA. The most promising results were obtained for the salts with imidazole and 1-methylimidazole co-formers.

## 1. Introduction

The discovery of new active pharmaceutical ingredients (APIs) having poor water-solubility and bioavailability has become prevalent during the drug development process [1,2,3]. The responsibility for maintaining a consistent bioavailability throughout product life has led to regulatory requirements for polymorphism-screening experiments, which is described in the ICH Q6A (Specification: Test Procedures and Acceptance Criteria for New Substances and New Drug Products: Chemical Substances) [4]. Furthermore, the pharmaceutical industry has also been interested in using crystal engineering principles to prepare new multicomponent API solid forms based on the intermolecular interactions established between the APIs and a general recognized as safe co-former [5,6,7,8]. This strategy allows the improvement of the APIs’ efficacy; enhancing their physicochemical properties without changing their pharmacological behavior [6].

The accurate study of the intermolecular interactions opens the possibility to understand API physicochemical behavior and that of their multicomponent crystal forms [7,8]. For that, structural information is needed and is conventionally obtained from X-ray diffraction. The particular use of powder X-ray diffraction (PXRD) for structure determination of inorganic/organic and pharmaceutical systems has been exploited in several studies [1,9,10,11,12,13,14,15]. This approach revealed to be a powerful tool, especially for systems in which the crystallization into single crystal is limited or difficult.

Depending on the position of the labile hydrogen atom, engaged in the hydrogen bonds, API solid forms can be defined as co-crystals or salts [16]. The distinction between the two is an important demand by the food and drug administration guidelines. To this end, Raman, solid-state NMR (ssNMR) and density functional theory (DFT) calculations have been used as complementary techniques to help locating the position of light atoms, such as hydrogens, and study packing interactions such as hydrogen bonds (HBs) [17,18,19]. Here, we use this powerful combination, not only for structural validation, but also for accurately study the HB packing interactions and relate them with some physicochemical properties. 

**Figure 1:**
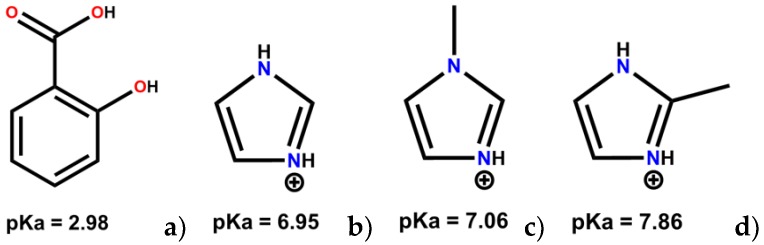
Molecular representation of protonated (**a**) SA; (**b**) IMI; (**c**) 1-MEIM and (**d**) 2-MEIM with their pKa values.

Salicylic acid (SA, Figure 1a) is a phenolic molecule also known as ortho-hydroxybenzoic acid or 2-hydroxybenzoic acid. This compound has antiseptic, preservative, analgesic and anti-inflammatory properties, covering a broad spectrum of applications, including skin-care products [20,21]. SA as free acid has a very low solubility in water (2.17 mg/mL) and it is normally administrated as sodium salt [21]. However, this form is not well regarded, as it exhibits some toxicity and has the tendency to cause gastrointestinal irritation [22]. To surpass the SA solubility issues, Kawashima and co-workers developed a strategy consisting on spray drying SA, dispersed in acacia solutions, improving its solubility by 50% [23]. Furthermore, the dissolution rate was also improved, being about 60 times faster than pure SA. Those results were related not only to the acacia concentration, but also to the amount of amorphous material in the spray-dried product. Ananikov and co-workers also reported some increases in SA solubility through the preparation of ionic liquids based on alkyl imidazole derivatives [24].

In the last decade, the propensity of SA to produce multicomponent crystal forms was extensively studied and the results showed a tendency to obtain ring synthons, when co-crystals are obtained, and different HB patterns upon salts formation [25,26,27,28,29,30,31,32,33,34,35,36,37,38,39,40,41]. Only for one of the reported studies, the SA solubility was slightly improved when co-crystallized with ethenzamide [42]. Until today, no other publications reporting the synthesis of SA co-crystals/salts to improve physicochemical/biological properties were published. Taking this into account and considering the low SA aqueous solubility, as well as the limitations of the available sodium form, we present herein the synthesis and characterization of three new SA molecular salts with improved solubility and dissolution rate properties. For this purpose, SA was combined with imidazole (IMI) and two derivatives, 1- and 2-methylimidazole (1-MEIM and 2-MEIM) (Figure 1). Imidazoles are ubiquitous in nature (e.g., amino acids) and play a critical role in many structures and functions within the human body [43]. Furthermore, their derivatives (e.g., 1-alkyl imidazoles) and salts are known to be biocompatible, exhibiting antimicrobial and antifungal activities, which make them good co-former candidates for the preparation of multicomponent pharmaceutical forms [44]. 

## 2. Results and Discussion

In this work, we intended to obtain new multicomponent crystal forms of SA, so co-former selection was based in its biological safety, capacity to participate in HB interactions and ΔpKa rule (if ΔpKa ≥ 3 salts are obtained, for ΔpKa ≤ 1 co-crystals) [45]. Three new salts were prepared mechanochemically through the milling of SA with IMI, 1-MEIM and 2-MEIM, under room temperature conditions and in absence of solvents. All reactions were completed, as no trace of starting materials were observed in the final products. Since the crystallization of these salts into single crystals was difficult and considering that solution synthesis normally requires large quantity of solvent and more time to be conducted, mechanochemistry is used as a reliable powerful procedure for the preparation of pharmaceutical salts and co-crystals with high purities and yields. 

The packing features of the three new SA salts, together with their dissolution rate, solubility and thermal stability properties, are presented and thoroughly discussed. Furthermore, a combination of PXRD with Raman, ssNMR and DFT calculations were performed for all compounds in order to confirm and validate the salt formation.

### 2.1. Structural Analysis

**Figure 2:**
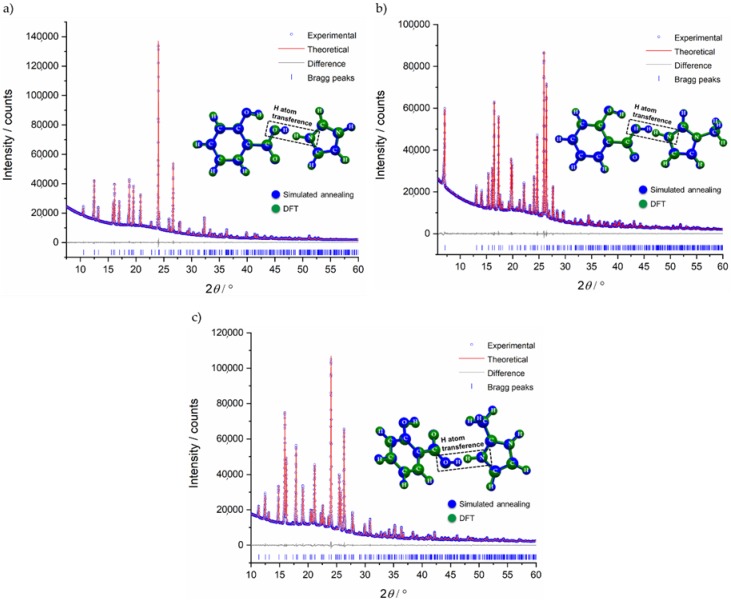
Rietveld refinement plot for (**a**) SA:IMI, (**b**) SA:1-MEIM and (**c**) SA:2-MEIM, displaying the experimental powder pattern (blue), the calculated powder pattern (red), the difference curve (grey), and the reflection positions (vertical blue dashes). A superposition of simulated annealing and density functional theory (DFT) structures is also depicted in blue and green, respectively.

The powdered samples were indexed as orthorhombic unit cells, using both TOPAS [46,47] and DICVOL [48] in DASH [49] program without ambiguity (see Table 1). Using Hofmann’s volume increments [50], the expected molecular volume was 247.3 Å^3^ and 271.3 Å^3^ for SA:IMI and SA:1-MEIM/SA:2-MEIM, respectively. Those volumes corresponds to 8 molecules in the unit cell (Z = 8) for a unit cell volume varying between 2019.92 Å^3^ and 2239.1 Å^3^ (Table 1). As starting configuration models, SA, IMI, 1-MEIM and 2-MEIM were taken from the CSD (see experimental section) and used in the simulated annealing process (real space method) to solve all structures. These structures were then refined by the Rietveld method (Figure 2). For the best final models, an optimization of hydrogen and non-hydrogen atoms was performed by DFT calculations. Results show that the models obtained by simulated annealing are correct and suggest more stable structures in which the labile proton, initially located in the SA carboxylic acid group, migrates to the nitrogen atom of all co-former molecules (IMI, 1-MEIM and 2-MEIM), forming 1:1 molecular salts. Raman data (Figures S1–S4) confirm the salts formation, as the SA C-O (COOH) vibrational band at 1635.9 cm^−1^, shifts towards values between 1629.5 cm^−1^ and 1627.2 cm^−1^, with a remarkable decrease in the intensity, supporting the presence of carboxylate groups. Furthermore, these results are also in agreement with the ΔpKa rule (3.97–4.88), which supports the salt formation.

**Figure 3:**
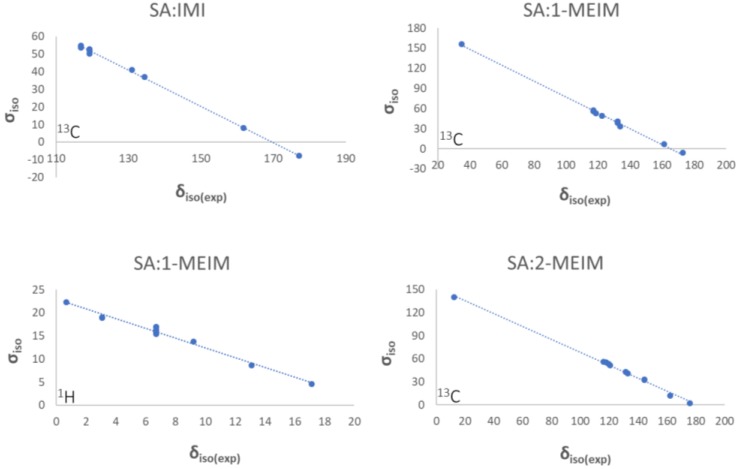
Correlations between theoretical chemical shieldings (σ_iso_), obtained from periodic plane-wave density functional theory calculations, against experimental ^1^H and ^13^C δ_iso(exp)_ of SA:IMI, SA:1-MEIM and SA:2-MEIM.

Unambiguous assignment of ^13^C ssNMR resonances was achieved by plotting the theoretical chemical shielding (σ_iso_) against the experimental CSs (δ_iso(exp)_) (Figure 3). As can be observed, there is a good correlation between both σ_iso_ and δ_iso(exp)_ variables, indicating that the models solved by PXRD are reliable. In the case of SA:1-MEIM, it was possible to assign the ^1^H resonances as the signal resolution was good enough. For SA:IMI and SA:2-MEIM (Figures S5 and S9), such assignment was not possible due to the very poor resolution of ^1^H NMR spectra. All ssNMR spectra are presented in Figures S5–S10.

**Figure 4:**
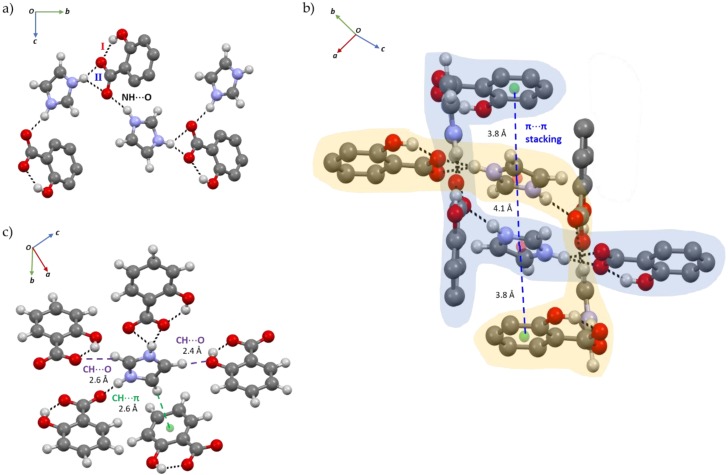
(**a**) Chain of hydrogen bond (HB) interactions between SA and IMI; (**b**) Packing interactions evidencing the channel formed between two crossed-chains and supported by π-π interactions; (**c**) Packing structure evidencing the weak C-H_(IMI)_⋯π_(SA)_ and C-H_(IMI)_⋯O_(SA)_ HB interactions. SA aliphatic hydrogen atoms were omitted for clarity.

The HB network of SA:IMI is based on an intramolecular S(6) O-H_(SA)_…O^−^_(SA)_, d_H_^…^_O_ = 1.76(8) Å, intermolecular N-H_(IMI)_⋯O^−^_(SA)_, d_H_^…^_O_ = 1.78(7) Å (mode I) and bifurcated ^+^N-H_(IMI)_⋯O^−^_(SA)_, d_H_^…^_O_ = 1.82(7) and 2.35(7) Å (mode II) HB interactions, which enable the formation of a chain composed by alternated SA:IMI molecules (Figure 4a). In the full packing structure, the presence of a channel formed by two independent crossed-chains is evident and supported by D_6h_ π-π interactions between SA and IMI molecules, d_π_^…^_π_ = 3.8 and 4.1 Å (Figure 4b). Furthermore, additional weak C-H_(IMI)_⋯π_(SA)_, d_H_^…^_π_ = 2.6 Å (in T-shaped form, C_2v_) and C-H_(IMI)_⋯O_(SA)_, d_H_^…^_O_ = 2.4 Å and 2.6 Å intermolecular interactions act as a bridge in the interconnection between the chains (Figure 4c).

**Figure 5:**
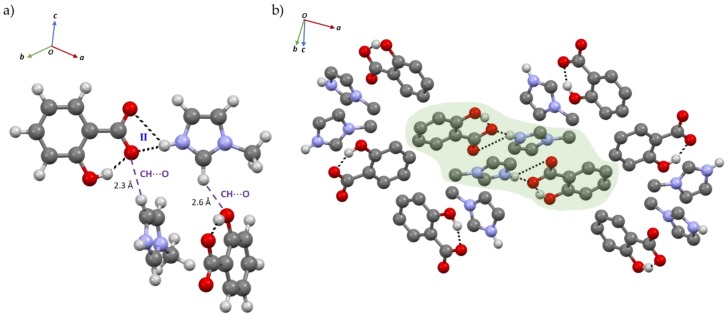
(**a**) Strong N-H_(1-MEIM)_⋯O_(SA)_ and weak C-H_(1-MEIM)_⋯O_(SA)_ HB interactions of SA:1-MEIM salt; (**b**) Packing interactions (light green) highlighting the two independent parallel layers. SA aliphatic hydrogen atoms were omitted for clarity.

**Figure 6:**
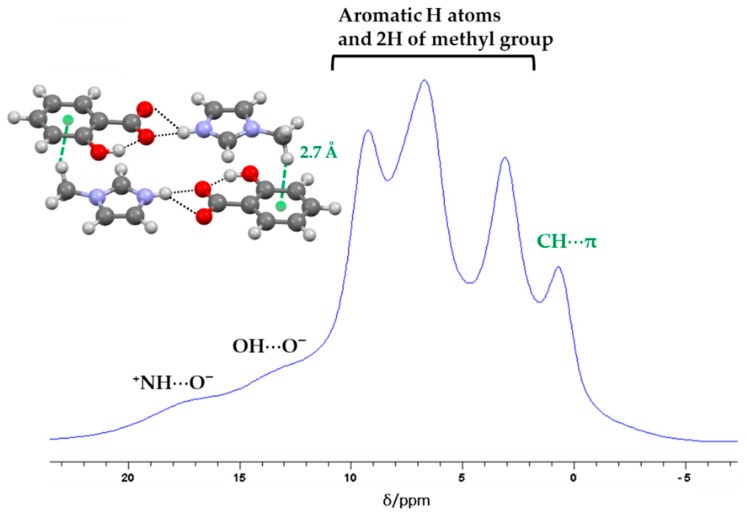
^1^H MAS NMR spectrum of SA:1-MEIM with signal attribution obtained from the correlation between δ_iso(exp)_ and σ_iso_. See experimental section for acquisition details and supplementary materials. SA:1-MEIM layer is also displayed, highlighting the weak C-H_(IMI)_⋯π_(SA)_ interaction.

Contrarily to SA:IMI, the co-former used in the preparation of SA:1-MEIM has one of the HB acceptors (nitrogen atom) blocked by a methyl group. The packing structure is now breaking from crossed-chains to small independent layers (Figure 5b), composed by one SA and one 1-MEIM molecules which are interacting through N-H_(1-MEIM)_⋯O^−^_(SA)_, d_H_^…^_O_ = 1.72(7) Å HB, mode II (Figure 5). The weak C-H_(1-MEIM)_⋯O_(SA)_, d_H_^…^_O_ = 2.3 and 2.6 Å interaction plays a similar rule as described for the previous salt, helping the packing growing in all directions and allowing the interconnection between the layers. The stability of this interconnection occurs due to the additional C-H_(1-MEIM)_⋯π _(SA)_, d_H_^…^_π_ = 2.7 Å HB. This interaction can be easily detected by ^1^H ssNMR, as the δ_iso(exp)_ of one of the methyl group hydrogen atoms is shifted towards higher field in comparison with the other two (Figure 6 and Table S2). The direct effect of imidazole ring current gives electron density to the hydrogen atom, directly pointed to the middle of the ring, resulting in a shielded effect. The hydrogen atoms involved in the N-H_(1-MEIM)_⋯O^−^_(SA)_ and O-H_(1-MEIM)_⋯O^−^_(SA)_ HBs appear, as expected, at lower field due to the deshielded effect of the oxygen atom that participates in the HB interaction.

**Figure 7:**
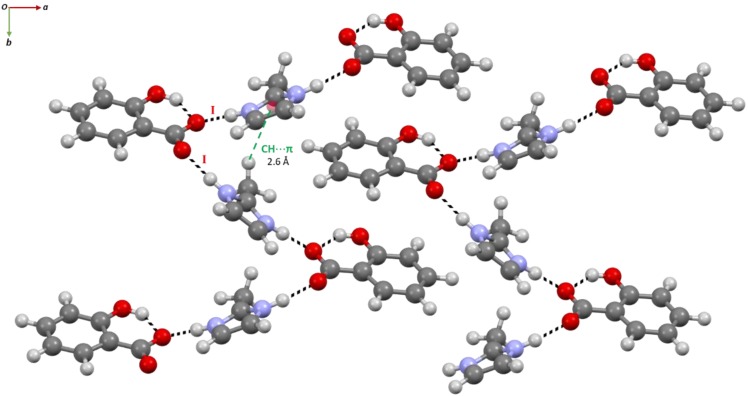
*Zig*-*zag* packing arrangement of alternated SA/2-MEIM molecules, along *b*, evidencing the ^+^N-H_(2-MEIM)_⋯O^−^_(SA)_ and C-H_(2-MEIM)_⋯π_(2-MEIM)_ HB interactions.

In SA:2-MEIM, the co-former methyl group changes from the nitrogen position to the carbon located between the nitrogen atoms of imidazole ring. The packing structure now evidences a similar type of HB connectivity like in SA:IMI, but with a different pattern (Figure 7). There is an intramolecular HB between the SA hydroxyl and carboxylate groups, which is never broken in all salts systems, and two independent HB interactions are established between two 2-MEIM molecules and one SA carboxylate group (mode I), ^+^N-H_(2-MEIM)_⋯O^−^_(SA)_, *d*_H_^…^_O_ = 1.72(7) Å and N-H_(2-MEIM)_⋯O^−^_(SA)_, *d*_H_^…^_O_ = 1.78(6) Å. Each 2-MEIM molecule has a different orientation (up and down) with respect to the plane formed by the SA molecule. The difference in the orientation is caused by the steric hindrance of the methyl group, forcing the molecules to deviate from each other. This orientation is also supported and stabilized by the weak C-H_(2-MEIM)_⋯π_(2-MEIM)_, *d*_H_^…^_π_ = 2.6 Å interaction established between the two 2-MEIM molecules. In the overall packing structure, the orientation of the strong SA^…^2-MEIM HB interactions induce the formation of intercalated *zig*-*zag* chains along *b*. 

### 2.2. Structure-Property Relationships

Dissolution rate, solubility and thermal stability studies were performed for all SA compounds and a tentative attempt to correlate the structures with the properties was undertaken. 

**Figure 8:**
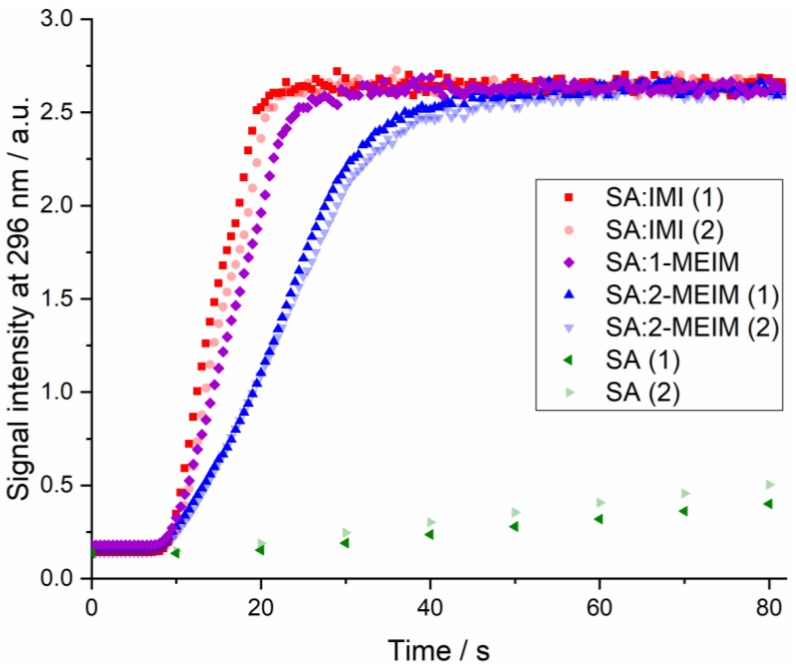
Dissolution rate plot of SA, SA:IMI, SA:1-MEIM and SA:2-MEIM, in both first (1) and second(2) assays, with intensity values of signal at 296 nm (SA absorption band) over relative time (addition of materials at 0 s).

Figure 8 shows the plot with the dissolution rate studies performed for both SA and SA salts. The duplicates for SA:IMI and SA:2-IMI show a very good correlation. A minor offset in the SA:IMI measurements might be caused by some uncertainty in time of addition, as the second tablet of SA:IMI was floating a short time before sliding down to the bottom of the beaker. Due to the high concentration of the samples, the UV-VIS spectra of all salts show saturation at the end of the study. As the aim of this experiment was to follow the dissolution rates of the materials compared to pure SA, only the first data points of the step are of interest and the fact of saturation at the end was considered as negligible.

As expected, all studied salts dissolve much faster than pure SA (Figure 8). This result can be somehow explained by the changes in the pH of the API micro-environment, during the dissolution process, such that more API dissolves in the diffusion layer that surrounds the particles, being faster dissipated into the bulk medium [51].

**Figure 9:**
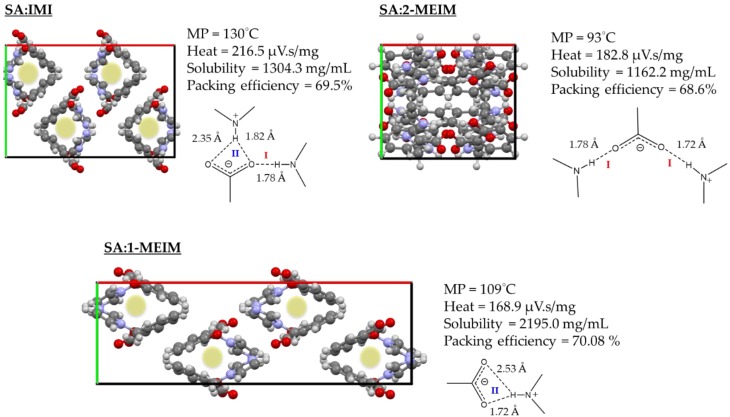
Overall packing view along *c* for SA:IMI, SA:1-MEIM and SA:2-MEIM alongside with details about melting point temperature (MP), heat, solubility, packing efficiency and HB interactions.

Dissolution rate is a complex physicochemical property and can be intimately related with several microscopic properties such as particle size, surface area, packing efficiency and different salt counter-ions [52]. In Figure 8 is visible that SA:IMI and SA:1-MEIM show very similar dissolution behavior, while SA:2-MEIM seems to dissolve more slowly. A possible explanation can be the different packing features. As can be observed in Figure 9, SA:IMI and SA:1-MEIM have similar molecular arrangements (along *b*), regardless of the different HB patterns previously discussed. The cavities observed along *b* (Figure 9, green highlight) can probably help in the water diffusion into the lattice, accelerating the dissolution process. In SA:2-MEIM, no cavities are observed (along all directions), despite having the lower packing efficiency (68.6%), which in turn should give a higher dissolution rate in comparison to the other salts (Figure 9).

Preliminary solubility studies in water show a drastically improvement in SA solubility, with the following order: SA:2-MEIM < SA:IMI < SA:1-MEIM (Figure 9). This order is inverse compared to the co-formers solubility: 1-MEIM < IMI < 2-MEIM. SA:2-MEIM, which exhibits the lowest dissolution rate also has the lowest solubility. 

As SA belongs to class II of the biopharmaceutical classification system, increasing aqueous dissolution rate and solubility is one of the key factors for improving its bioavailability. In general, salts can stay in solution in a supersaturated state and might not precipitate immediately, even if the pH of the gastrointestinal tract favors the insoluble free acid formation. Supersaturation can leave, in fact, a wider time window for the API absorption. Nevertheless, when salt precipitation occurs, amorphous forms as well as fine particles are normally obtained, which have higher dissolution rate and also higher bioavailability [51].

Regarding the thermal stability, from DTA data (Figures S11–S13), all salts show a similar decomposition temperature (T_onset_ between 192 °C and 205 °C), a lower melting point than pure SA (approximately 158.6 °C) and absence of phase transitions or solvents. SA:IMI has a higher melting point than SA:1-MEIM and SA:2-MEIM, presenting the most stable lattice. Furthermore, the heat required to break the salts interactions, directly proportional to the ΔH_fus_, follows the order: SA:1-MEIM < SA:2-MEIM < SA:IMI. In Figure 9 are presented the main synthons observed for each SA salt. The charged assisted HB distances found in each system are very similar (Table 2) and the main difference is the type and number of connections, which can possibly influence the observed trend. For SA:IMI there are two strong HB interactions in two modes (I and II) and for SA:2-MEIM also two strong HB interactions, but in mode I. In addition, for SA:IMI, there are also several weak HB interactions (CH⋯O, CH⋯π and π- π), which together with the strong ones, helps to stabilize the structure. 

In SA:1-MEIM, only mode II is observed, but because the hydrogen atom is not in the center of the bifurcated interaction, one of the distances is much higher than the other one, and hence only one strong interaction is observed (1.72 Å), which can explain the lower heat energy required. 

## 3. Materials and Methods 

### 3.1. Synthesis

All reagents were purchase from Sigma Aldrich and used without further purification. SA:IMI, SA:1-MEIM and SA:2-MEIM were mechanochemically synthesized using a vibratory ball mill (Retsch MM400 horizontal mill, Haan, Germany). SA and co-formers were weighted into a stainless-steel vessel (milling jar 10 mL) in an equimolar ratio with a total mass of 1 g. Mixtures were ground using two stainless-steel balls (*ϕ* 10 mm, 4 g) at 30 Hz for 25 min, under room temperature conditions (25 °C, 55% humidity) and in absence of solvents.

### 3.2. Powder X-Ray Diffraction (PXRD)

Data collection was performed at room temperature on a D8 Discover diffractometer (Bruker AXS, Karlsruhe, Germany), using Cu radiation (Cu Kα 1, λ = 1.5406 Å), a Johansson monochromator, and a position-sensitive LYNXEYE detector. Sample was contained in a rotating glass capillary with 0.5 mm diameter, mounted horizontally and spun at 60 min^−1^ for minimizing preferred orientation. Data were collected in a 2*θ* from 5 to 60° with a step size of 0.009 and 9 s per step. 

Powder patterns were indexed using DICVOL [4,8] in DASH [49] and TOPAS [46,47]. Structure solution was achieved by the real-space method, using simulated annealing routine implemented in DASH [49]. The starting models of SA, IMI, 1-MEIM and 2-MEIM molecules were obtained from the CIF files of CSD (Cambridge Structural Database) with the entries SALIAC [53], IMAZOL01 [54], BARMIM [55] (only the 1-MEIM structure) and FULPIM [56], respectively. During simulated annealing no restrictions in the degrees of freedom of each molecule was applied.

Rietveld refinement was carried out using TOPAS [46,47] for the full 2*θ* range. A Pawley fit was first performed and in the following refinement steps, the scale factor, background, atomic positions, and isotropic displacement parameters were refined.

For all structures, two different isotropic displacement parameters were refined: one for the non-hydrogen (non-H) atoms of each SA and IMI, 1-MEIM and 2-MEIM molecules, and another one for the hydrogen atoms of both SA and IMI, 1-MEIM and 2-MEIM, B_iso_(H) = 1.2 × B_iso_(non-H). Restraints were applied for all bond lengths, angles, and planar rings/groups. Data collection and structure refinement details are summarized in Table 1. All information about HB distances and angles for all compounds, obtained using PLATON [57], are presented in Table 2. CCDC 1958159-1958161 contain the supplementary crystallographic data for this paper. These data can be obtained free of charge from The Cambridge Crystallographic Data Centre via www.ccdc.cam.ac.uk/structures.

### 3.3. Density Functional Theory (DFT) Calculations

DFT calculations were performed with Quantum Espresso (QE) package using the Perdew-Burke-Ernzerhof exchange-correlation function [58]. The molecular models were obtained by the periodic expansion of the crystallographic unit cells for SA:IMI, SA:1-MEIM and SA:2-MEIM systems. The computational procedure consisted in a first optimization of the atomic positions, followed by calculation of the NMR parameters using the GIPAW approach [59]. The pseudopotentials of the norm-conserving Troullier-Martins [60] type with GIPAW [59] reconstruction were taken from other references [61,62]. The cutoff energy for the plane waves was set as 30 Ryd, and Monkhorst-Pack *k*-points grids of 1 × 2 × 2 (SA:IMI), 1 × 2 × 2 (SA:1-MEIM) and 2 × 2 × 1 (SA:2-MEIM) were employed. QE default values for convergence criteria were used. The calculated ^13^C isotropic σ_iso_ were plotted as a function of the δ_iso(exp)_. For SA:1-MEIM, a plot with the ^1^H σ_iso_ and δ_iso(exp)_ values were performed as the experimental data had enough resolution for signal attribution. Reference values were obtained from the equation plots traced for each salt system (Figure 2). Conversion of the calculated σ_iso_ into the corresponding δ_iso(DFT)_ was performed according to

δ_iso(DFT)_ = (σ_iso_ − σ_ref_)/*m* (1)

where σ_ref_ and *m* are the intercept and slope of the regression model, respectively, obtained from the best fit.

### 3.4. Solid-State Nuclear Magnetic Resonance (ssNMR)

Magic angle spinning (MAS) solid-state NMR spectra (Figures S5–S10) were acquired on a Bruker Avance 400 spectrometer operating at a *B*_0_ field of 9.4 T with Larmor frequencies of 400.1 MHz and 100.6 MHz for ^1^H and ^13^C, respectively. All ^1^H and ^13^C NMR experiments were recorded using a double-resonance 4 mm Bruker MAS probe at a spinning rate of 10 KHz. The isotropic chemical shift values are given with respect to TMS (0 ppm) using adamantane as secondary standard for ^1^H and ^13^C measurements.

^1^H-MAS studies were made with a π/2 pulse lengths of 3.2 μs, a recycle delay of 5 s and 16 accumulations.

The ^1^H-^13^C CPMAS NMR spectra were acquired using a contact time of 1 ms and a recycle delay of 5 s. The accumulation number (ns) is given in the caption of the respective figure.

### 3.5. Thermal Stability Studies

Thermal gravimetric analyses (TG) and differential thermal analyses (DTA) were simultaneously recorded by a thermobalance SETARAM TAG 24. Measurements were conducted in flowing nitrogen (≈35 × 10^−5^ L/s) after repeated evacuation (≈3 × 10^−2^ mbar) on powdered samples placed in open platinum crucibles (100 µL) at a heat rating of 10 K/min up to a maximum temperature of 300 °C. Cooling to room temperature was carried out at −30 K/min followed by one repetition of this heating and cooling procedure. For correction of buoyancy effect the data of the second run were subtracted from the first run. The sample mass was recorded with an accuracy of ± 0.01 mg.

### 3.6. Solubility Studies

Preliminary solubility studies were carried out by dissolving ≈10 mg of each molecular salt in water, gradually added until reaching complete dissolution and saturation. The amount of added water allowed the determination of empirical solubility values and SA was used for comparison. Table 3 shows the results obtained.

### 3.7. Dissolution Rate Studies

Samples were compressed to tablets with a hydraulic press to ensure reproducible conditions. These tablets were dropped in a round-bottom glass beaker filled with 60 mL of deionized water and equipped with a motor driven PTFE centrifugal stirrer shaft running at 500 rpm (EURO-ST P CV, IKA-Werke GmbH & Co. KG., Staufen, Germany). The solution was pumped to the UV-VIS flow cell (Z-geometry, self-made construction, PEEK, 1 mm optical path) with a peristaltic pump (Ismatec Reglo Analog, Cole-Parmer GmbH, Wertheim, Germany) at a pump speed setting of 75%, which equals a flow rate of 7.4 mL/min. The measurements were done using a UV-VIS spectrometer (MultiSpec Pro, tec 5 AG, Oberursel, Germany) acquiring a spectral range of 190–720 nm with a sampling rate of 500 ms for SA salts and 10 s for pure SA. Absorbance data was collected as ASCII, combined using a python script and evaluated by univariate monitoring of the maximum intensity at 296 nm. Data was plotted for comparison using Origin (Origin 2018G, OriginLab Corporation, Northhampton, MA, USA).

**Figure 10:**
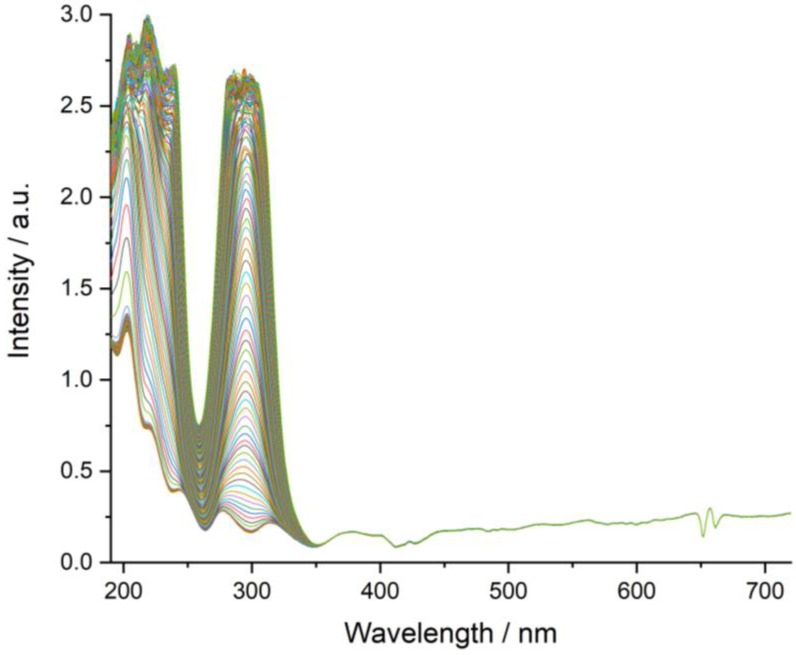
UV/VIS spectra of SA:2-MEIM dissolution study stacked over time, showing the building-up of signals in the range of 200–300 nm. A total of 252 spectra were acquired with a sampling rate of 500 ms.

A total of five SA salts samples, as well as two samples of pure SA were studied with the UV-VIS setup. The measurements on SA:IMI and SA:2-MEIM were performed in duplicates. Due to some issues with compressing the sample of SA:1-MEIM there was only a single tablet available of this material. As an example, the acquired spectra of SA:2-MEIM (1^st^ assay), Figure 10, clearly shows the build-up of signals in the UV-range from 200–350 nm. For the univariate data approach, the well-separated signal at 296 nm was chosen for intensity evaluation.

## 4. Conclusions

Three novel imidazole-based salts of SA were mechanochemically prepared and their structural characterization performed using PXRD combined with spectroscopic techniques. Analysis of ^1^H and ^13^C-CPMAS NMR data along with Raman spectra and DFT calculations was helpful in determining the deprotonation state of SA carboxylic group, allowing unambiguous identification of salt forms. The supramolecular arrangement of all molecular salts is based on strong ^+^N-H_(SA)_⋯O^-^_(co-former)_ HB interactions and the full packing changes depending upon the presence and position of the co-former methyl group. The packing structure of SA:IMI is based on independent crossed-chains and, when the co-former has one of the HB donor position blocked by a methyl group, the packing changes being mainly composed by small independent layers. Interestingly, in a perspective along *c*, both packing structures present similar small cavities. In this case, the face to face methyl group position in 1-MEIM substitutes the normal strong HB interaction between SA and IMI, producing the same visual effect. In the case of SA:2-MEIM, the methyl group located between the imidazole nitrogen atoms, now unblock the nitrogen donor position, being in a similar situation observed for SA:IMI. However, due to the steric hindrance induced by the methyl group, the two 2-MEIM co-formers, which are interacting with the SA carboxylate group, deviate from each other, being positioned above and below the planar ring of SA, thus helping in the formation of zig-zag chains.

Physicochemical studies show that all salts present much higher solubility and faster dissolution rate than SA as free acid. Since the dissolution rate also depends on the structure packing, in our results there is an apparent relation between the two properties for the case of SA:IMI and SA:1-MEIM. Both structures present cavities along *c*, which we believe to help water to diffuse into the lattice. This somehow explains why they present similar dissolution rate profiles. In terms of thermal stability, all salts present lower melting points than SA free acid. The strength of the HBs is similar for all salts and the difference lies in the number and type of interactions. SA:IMI has more interactions (strong and weak, type I and II) than the other salts and hence is more stable, presenting both highest melting point and heat. The results herein presented show that all SA salts are promising candidates to be used as new solid forms for SA delivering.

## Figures and Tables

**Table 1:** Crystallographic details for salicylic acid (SA) salts.

	SA:IMI	SA:1-MEIM	SA:2-MEIM
Chemical formula	C_10_H_10_N_2_O_3_	C_11_H_12_N_2_O_3_	C_11_H_12_N_2_O_3_
Formula weight/g·mol^−1^	206.2	220.23	220.23
Crystal system	Orthorhombic	Orthorhombic	Orthorhombic
Space group	*Pbca*	*Pbca*	*Pbcn*
*a*/Å	16.6736(3)	24.9283(3)	10.8972(6)
*b*/Å	11.08182(19)	8.00291(6)	9.2671(5)
c/Å	10.9319(2)	10.98695(11)	22.1726(11)
*V*/Å^3^	2019.92(6)	2191.89(4)	2239.10(2)
*Z*	8	8	8
*R_p_*_,_*R^’^_p_*/_%_^1^	1.097, 10.065	1.179, 7.251	1.569, 7.136
*R*_wp,_*R^’^_wp_*/_%_^1^	1.514, 6.427	1.584, 5.495	2.056, 6.371
*R_exp_*_,_*R^’^_exp_*/_%_^1^	1.126, 4.779	1.067, 3.701	1.105, 3.234
*R_Bragg_*	0.70378	0.57544	0.81733
Gof	1.345	1.485	1.752

^1^ Dashed values corresponds to values after background subtraction.

**Table 2:** Hydrogen-bond distances and angles for the reported crystal salts.

Structure	sym op	D-H⋯A	*d*(D-H) (Å)	d(H⋯A) (Å)	d(D⋯A) (Å)	DHA (deg)
SA:IMI	x, y, z x, 3/2 − y, −1/2 + z 2 − x, 1 − y, 1 − z 2 − x, 1 − y, 1 − z	O-H_(SA)_⋯O^−^_(SA)_ N-H_(IMI)_⋯O^−^_(SA)_ ^+^N-H_(IMI)_⋯O^−^_(SA)_ ^+^N-H_(IMI)_⋯O^−^_(SA)_	0.91(7) 0.94(7) 0.94(7) 0.94(7)	1.76(8) 1.78(7) 2.35(7) 1.82(7)	2.525(9) 2.698 (12) 3.093(10) 2.733(11)	140(7) 163(6) 135(5) 161(6)
SA:1-MEIM	x, y, z x, y, z x, y, z	O-H_(SA)_⋯O^−^_(SA)_ N-H_(IMI)_⋯O^−^_(SA)_ ^+^N-H _(1-MEIM)_⋯O^−^_(SA)_	0.93(8) 0.93(7) 0.93(7)	1.65(7) 2.53(7) 1.72(7)	2.523(10) 3.128(13) 2.649(14)	154(7) 122(6) 176(8)
SA:2-MEIM	x, y, z 1/2 − x, 1/2 + y, z 1 + x, y, z	O-H_(SA)_⋯O^−^_(SA)_ N-H _(IMI)_⋯O^−^_(SA)_ ^+^N-H _(IMI)_⋯O^−^_(SA)_	0.93(7) 0.94(6) 0.94(7)	1.74(8) 1.78(6) 1.72(7)	2.551(10) 2.725(12) 2.654(10)	144(7) 176(8) 172(7)

**Table 3:** Preliminary solubility results obtained for SA, SA:IMI, SA:1-MEIM and SA:2-MEIM.

Compound	Solubility (mg/mL)
SA	2.38
SA:IMI	1304.3
SA:1-MEIM	2195.0
SA:2-MEIM	1162.2

## Supplementary Materials

The following are available online at https://www.mdpi.com/1420-3049/24/22/4144/s1, Table S1: Experimental and calculated ^13^C NMR CSs of SA:IMI, Table S2: Experimental and calculated ^1^H NMR CSs of SA:1-MEIM, Table S3: Experimental and calculated ^13^C NMR CSs of SA:1-MEIM, Table S4: Experimental and calculated ^13^C NMR CSs of SA:2-MEIM, Figure S1: Raman spectra of SA, Figure S2: Raman spectra of SA:IMI, Figure S3: Raman spectra of SA:1-MEIM, Figure S4: Raman spectra of SA:2-MEIM, Figure S5: ^1^H MAS NMR spectra of SA:IMI, Figure S6: ^13^C CP-MAS NMR spectra of SA:IMI, Figure S7: ^1^H MAS NMR spectra of SA:1-MEIM, Figure S8: ^13^C CP-MAS NMR spectra of SA:1-MEIM, Figure S9: ^1^H MAS NMR spectra of SA:2-MEIM, Figure S10: ^13^C CP-MAS NMR spectra of SA:2-MEIM, Figure S11: DTA curve of SA:IMI, Figure S12: DTA curve of SA:1-MEIM and Figure S13: DTA curve of SA:2-MEIM.
