# Supplementary material for: Insight into the Structure and Properties of Novel Imidazole-Based Salts of Salicylic Acid
Source: Molecules. 2019 Nov 15;24(22):4144. doi: 10.3390/molecules24224144 (PMC6891694; doi:10.3390/molecules24224144)
Supplement: Supplementary file 1 [file molecules-24-04144-s001.pdf]

Insight into the Structure and Properties of Novel Imidazole-Based Salts of Salicylic Acid

Inês C. B. Martins [1], Dominik Al-Sabbagh [1], Klas Meyer [1], Michael Maiwald,[1] Gudrun Scholz[2] and Franziska Emmerling [1,2*]

[1] Federal Institute for Materials Research and Testing (BAM), Richard Willstätter-Str-11, 10249 Berlin, Germany
[2] Department of Chemistry, Humboldt-Universität zu Berlin, Brook-Taylor-Str. 2, 12489 Berlin, Germany
* Correspondence: Franziska.Emmerling@bam.de; Tel.: +49 30 8104-1133

Supplementary Material

Table of contents

DFT results………………………………………………………….. 1
Raman data………………………………………………………… 2
ssNMR data…………………………………………………………. 3
DTA data…………………………………………………………… 7

Labeling structures

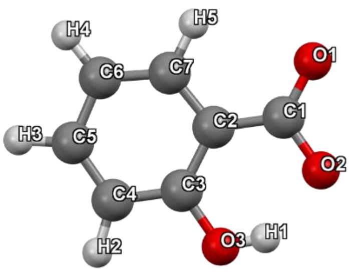

SA

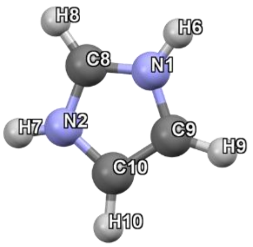

IMI

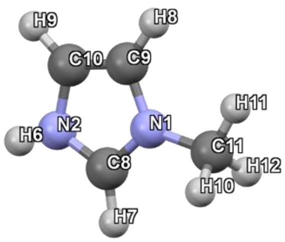

1-MEIM

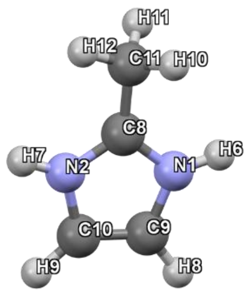

2-MEIM

DFT results

Table S1. Experimental and calculated ^{13}C NMR CSs of SA:IMI.

Atom label	$\delta_{iso(exp)}$/ppm	$\delta_{iso(DFT)}$/ppm	$\delta_{iso(exp)}-\delta_{iso(DFT)}$	$(\delta_{iso(exp)}-\delta_{iso(DFT)})^2$	RMSD
C1	177.1	177.4	-0.3	0.09	0.931
C2	161.7	162.2	-0.5	0.25	
C3	134.5	134.4	0.1	0.01	
C4	131.0	130.7	0.3	0.09	
C5	116.9	118.5	-1.6	2.56	
C6	116.9	118.2	-1.3	1.69	
C7	116.9	117.5	-0.6	0.36	
C8	119.4	119.3	0.1	0.01	
C9	119.3	120.1	-0.8	0.64	
C10	119.3	121.2	-1.9	3.61	

Table S2. Experimental and calculated ^{1}H NMR CSs of SA:1-MEIM.

Atom label	$\delta_{iso(exp)}$/ppm	$\delta_{iso(DFT)}$/ppm	$\delta_{iso(exp)}-\delta_{iso(DFT)}$	$(\delta_{iso(exp)}-\delta_{iso(DFT)})^2$	RMSD
H1	13.1	13.6	-0.5	0.25	0.339
H2	6.7	6.8	-0.07	0.005	
H3	6.7	6.3	0.4	0.16	
H4	6.7	5.7	1.03	1.06	
H5	6.7	5.7	1.03	1.06	
H6	17.1	17.4	-0.3	0.09	
H7	6.7	6.9	-0.2	0.04	
H8	9.2	8.7	0.5	0.25	
H9	6.7	7.1	-0.4	0.16	
H10	3.1	3.8	-0.7	0.49	
H11	3.1	3.8	-0.7	0.49	
H12	0.7	0.6	0.1	0.01	

Table S3. Experimental and calculated ^{13}C NMR CSs of SA:1-MEIM.

Atom label	$\delta_{iso(exp)}$/ppm	$\delta_{iso(DFT)}$/ppm	$\delta_{iso(exp)}-\delta_{iso(DFT)}$	$(\delta_{iso(exp)}-\delta_{iso(DFT)})^2$	RMSD
C1	172.9	171.2	1.7	2.89	1.085
C2	117.0	117.8	-0.8	0.64	
C3	161.1	159.9	1.2	1.44	
C4	117.0	117.4	-0.4	0.16	
C5	132.2	132.7	-0.5	0.25	
C6	117.0	116.8	0.2	0.04	
C7	132.2	131.7	0.5	0.25	
C8	133.9	135.0	-1.1	1.21	
C9	122.5	123.4	-0.9	0.81	
C10	118.8	120.1	-1.3	1.69	
C11	34.8	33.2	1.6	2.56	

Table S4. Experimental and calculated ^{13}C NMR CSs of SA:2-MEIM.

Atom label	$\delta_{iso(exp)}$/ppm	$\delta_{iso(DFT)}$/ppm	$\delta_{iso(exp)}$-$\delta_{iso(DFT)}$	$(\delta_{iso(exp)}-\delta_{iso(DFT)})^2$	RMSD
C1	177.1	178.7	-1.6	2.56	
C2	114.9	114.5	0.4	0.16	
C3	163.1	164.7	-1.6	2.56	
C4	116.6	115.4	1.2	1.44	
C5	144.4	142.7	1.7	2.89	
C6	119.4	117.9	1.5	2.25	1.100
C7	144.4	142.9	1.5	2.25	
C8	132.8	132.6	0.2	0.04	
C9	131.4	130.6	0.8	0.64	
C10	120.4	120.1	0.3	0.09	
C11	13.4	14.7	-1.3	1.69	

Raman data

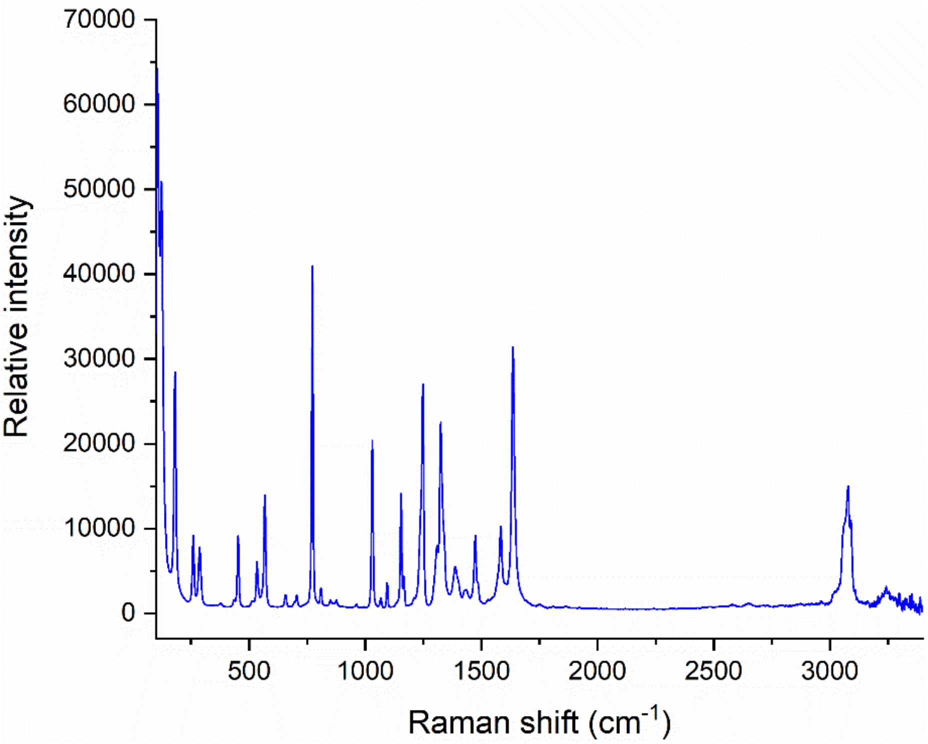

Figure S1. Raman spectra of SA.

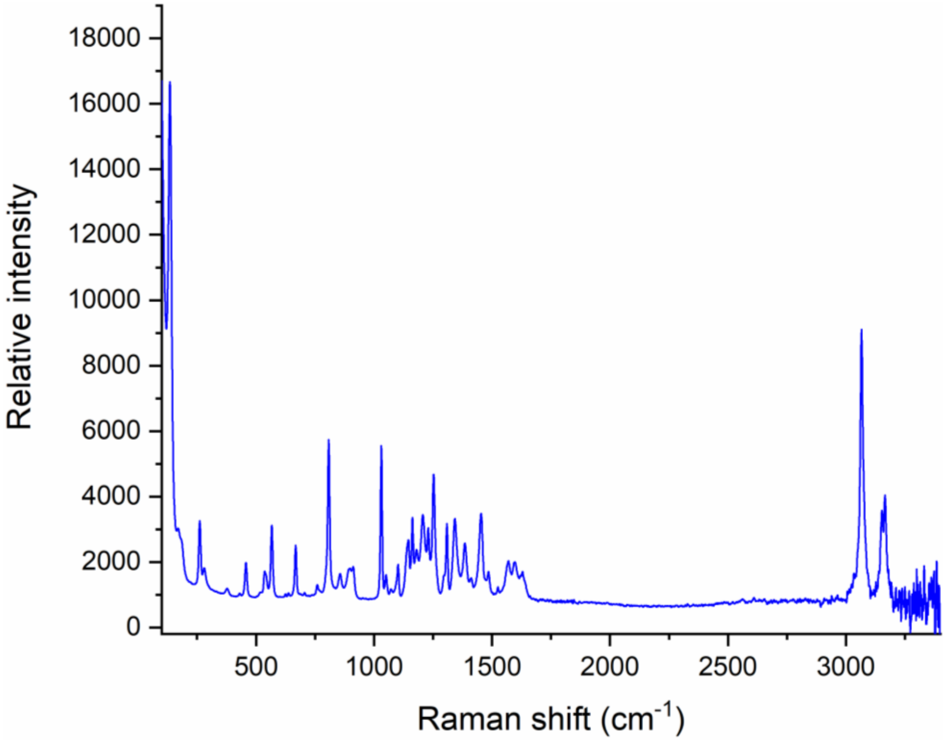

Figure S2. Raman spectra of SA:IMI.

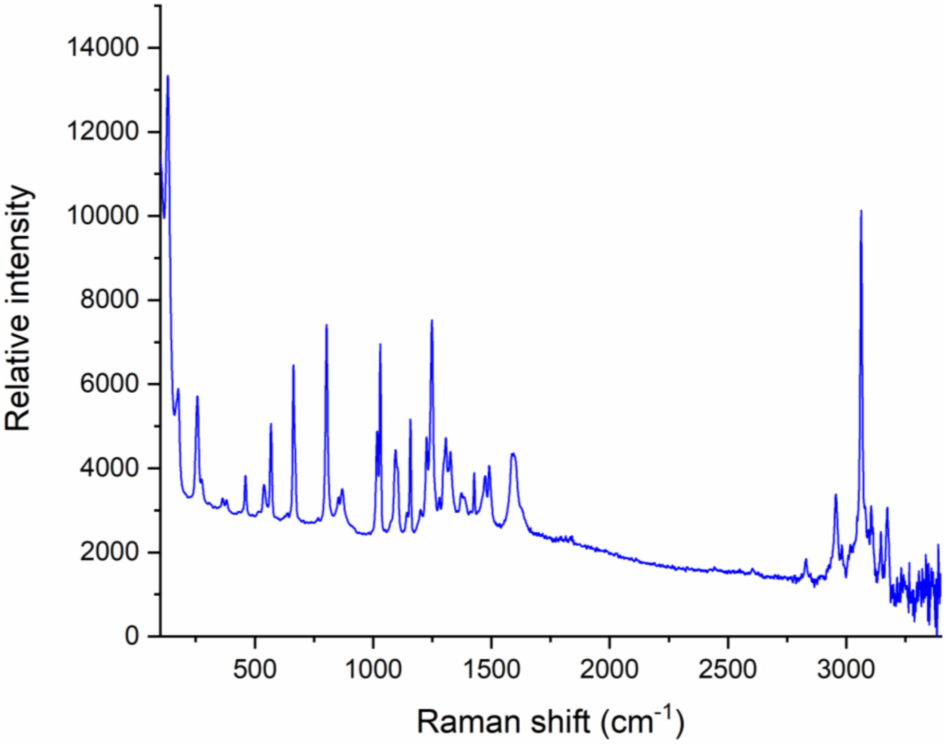

Figure S3. Raman spectra of SA:1-MEIM.

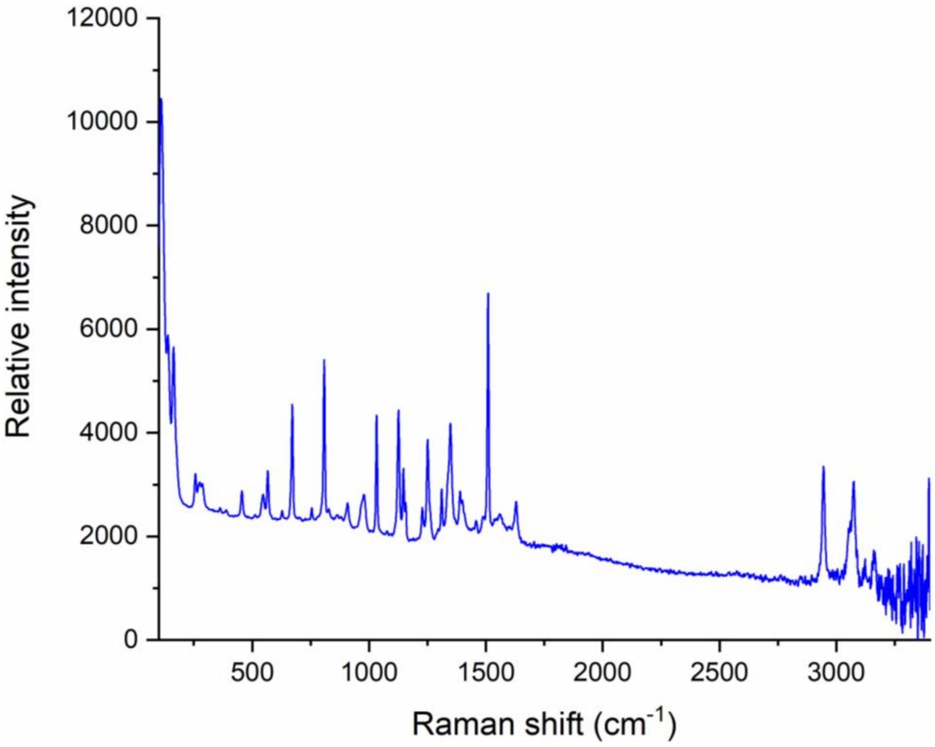

Figure S4. Raman spectra of SA:2-MEIM.

ssNMR data

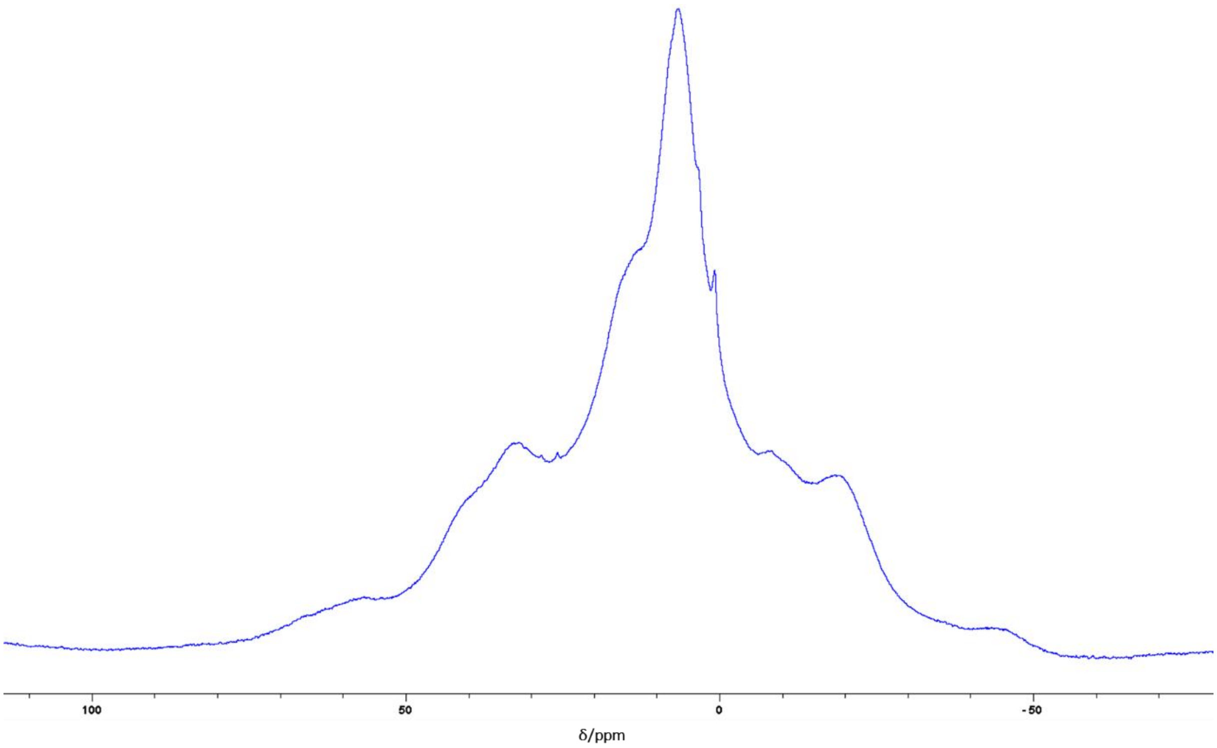

Figure S5. ^{1}H MAS NMR spectra of SA:IMI.

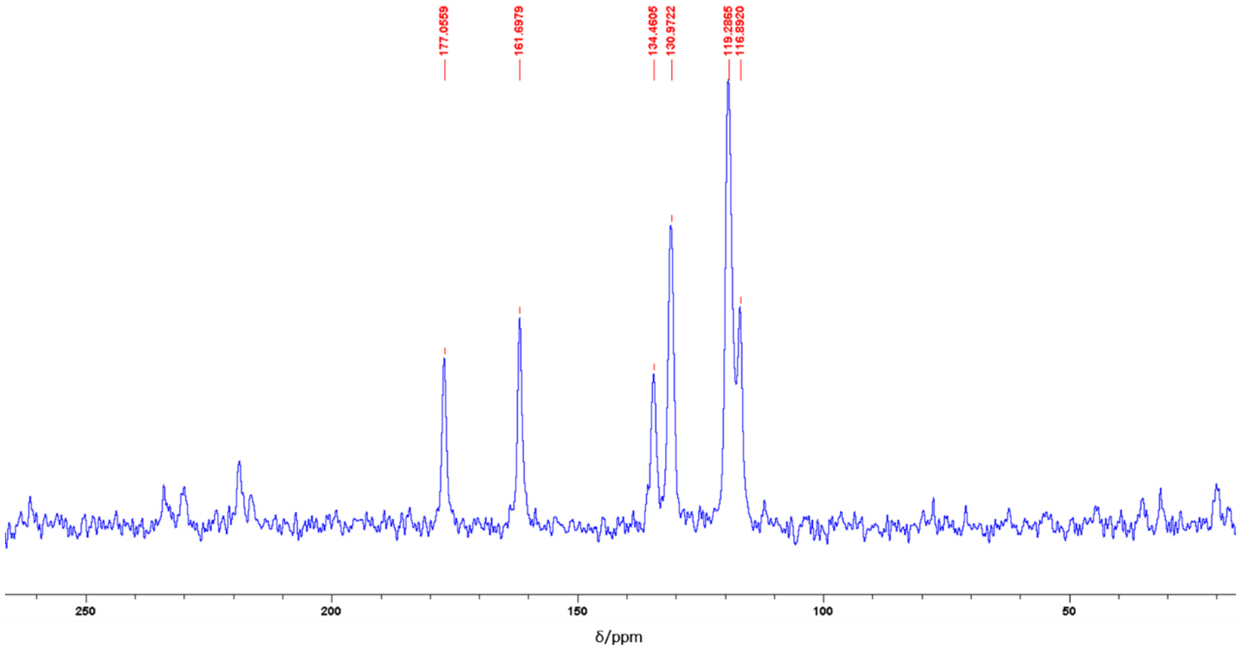

Figure S6. ^{13}C CP-MAS NMR spectra of SA:IMI.

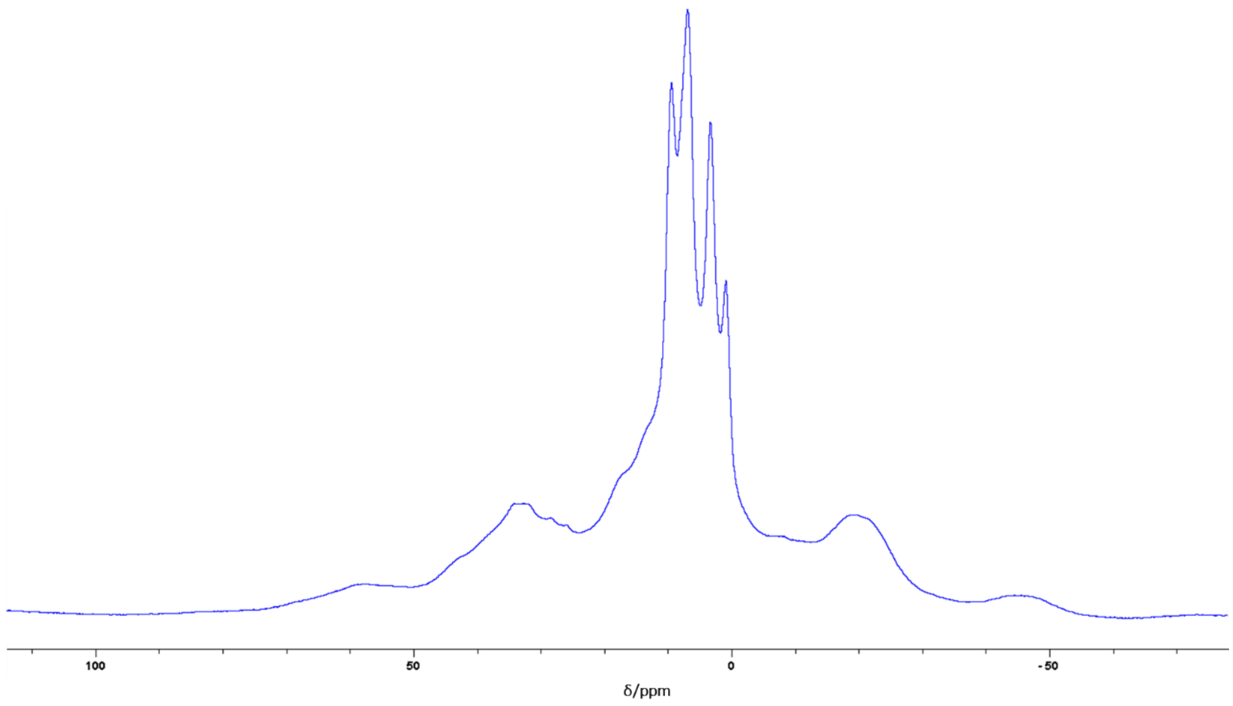

Figure S7. ^{1}H MAS NMR spectra of SA:1-MEIM.

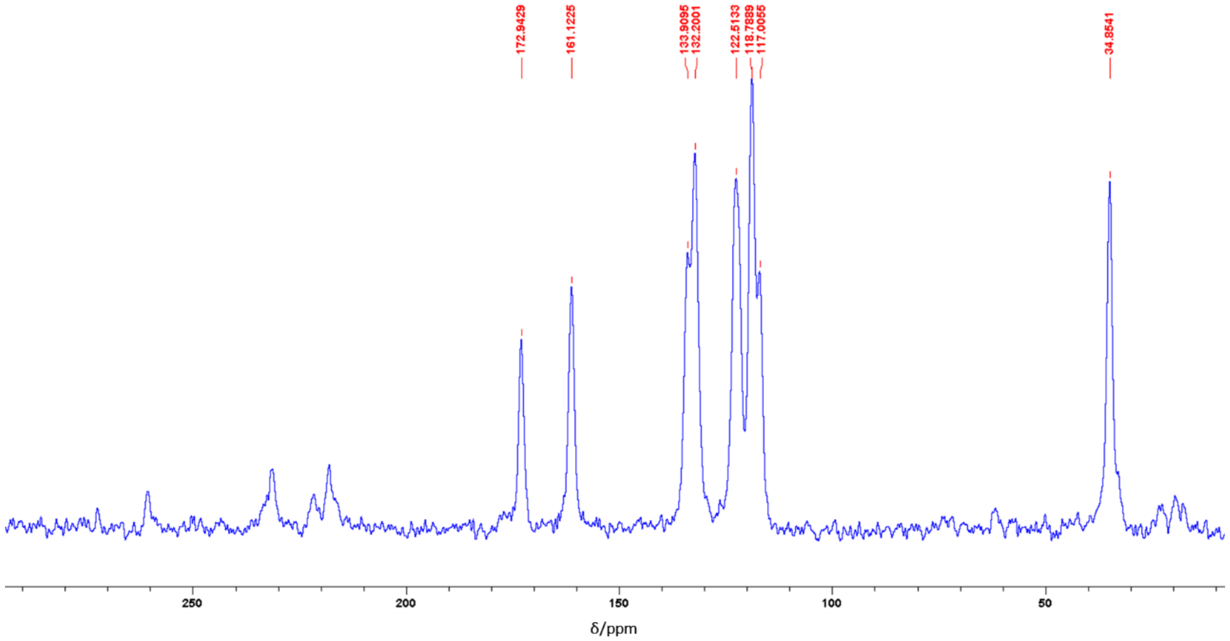

Figure S8. ^{13}C CP-MAS NMR spectra of SA:1-MEIM.

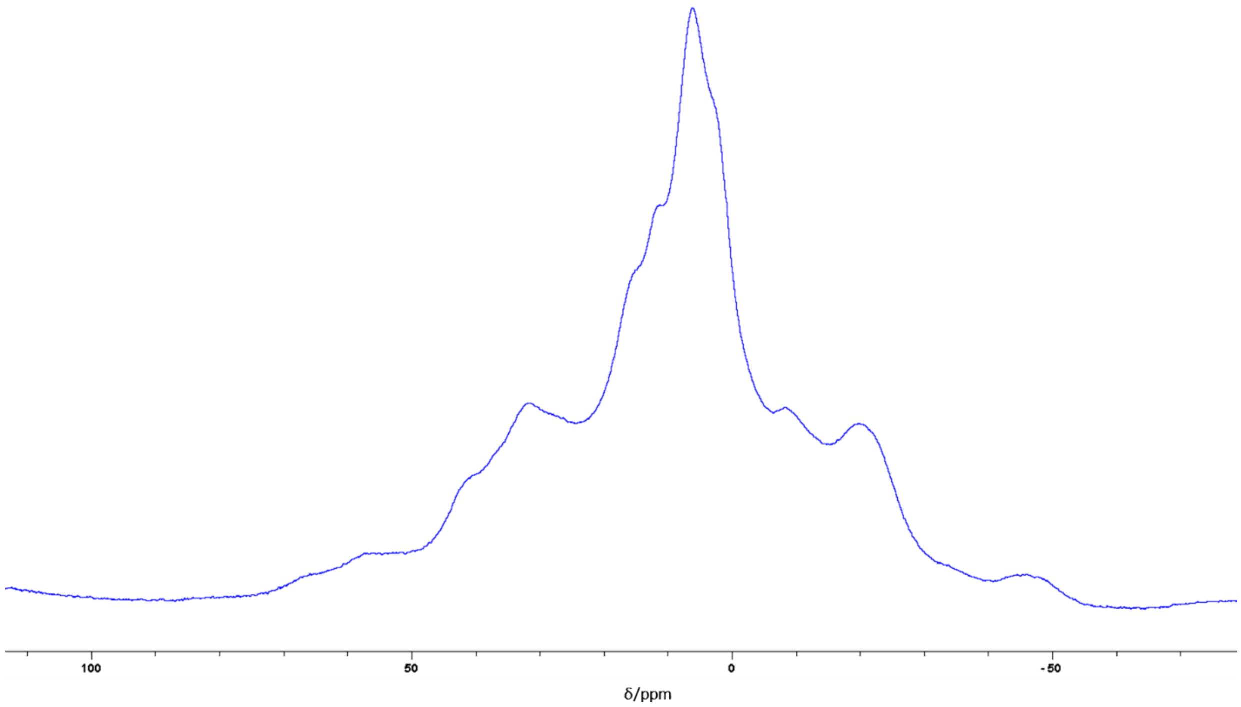

Figure S9. ^{1}H MAS NMR spectra of SA:2-MEIM.

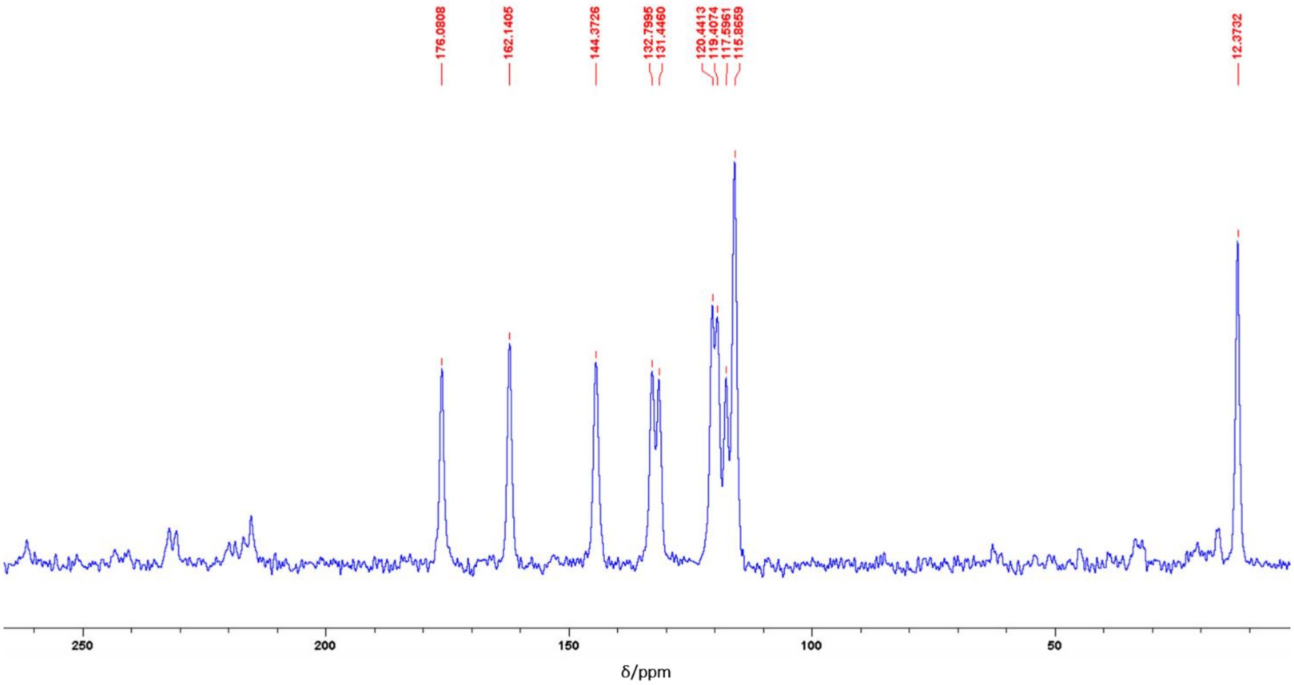

Figure S10. ^{13}C CP-MAS NMR spectra of SA:2-MEIM.

DTA data

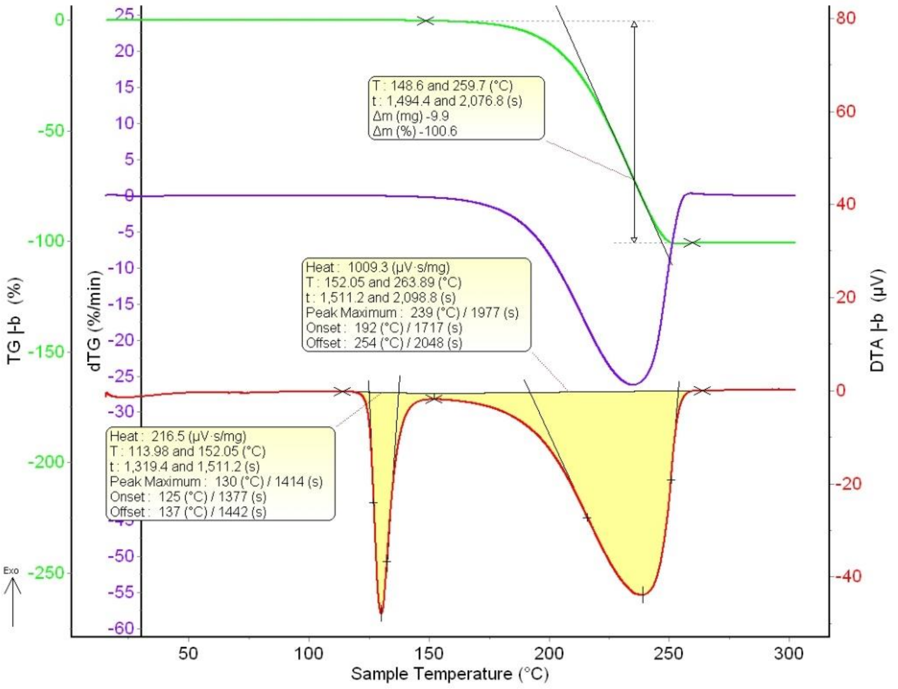

Figure S11. DTA curve of SA:IMI.

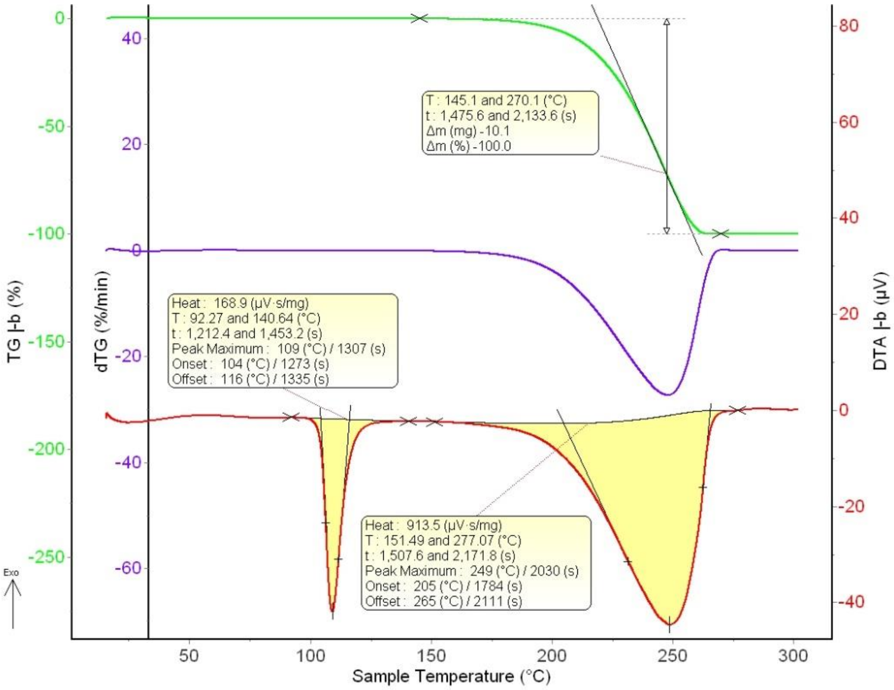

Figure S12. DTA curve of SA:1-MEIM.

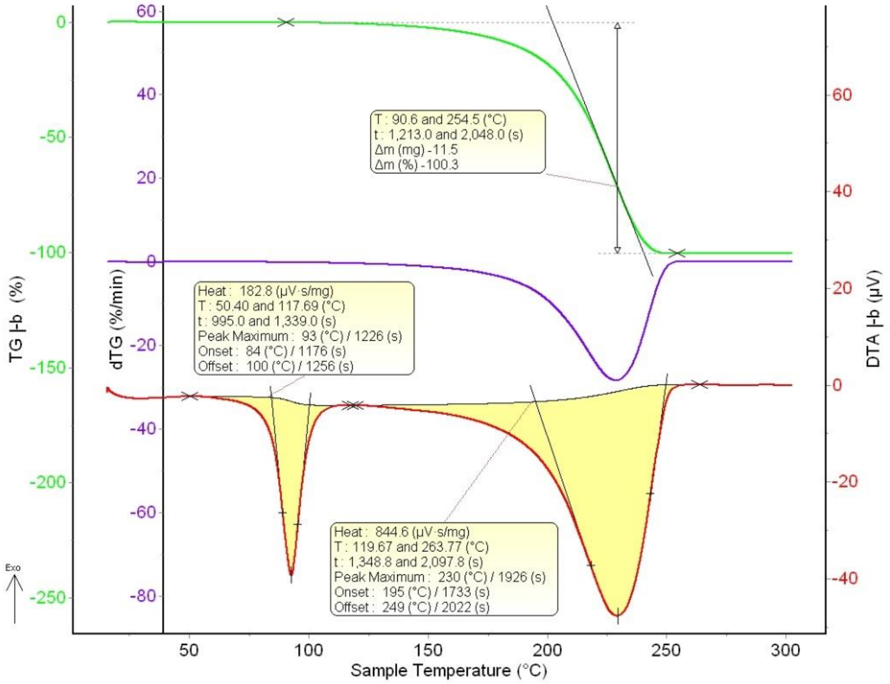

Figure S13. DTA curve of SA:2-MEIM.
